# Establishing the Breeding Provenance of a Temperate-Wintering North American Passerine, the Golden-Crowned Sparrow, Using Light-Level Geolocation

**DOI:** 10.1371/journal.pone.0034886

**Published:** 2012-04-10

**Authors:** Nathaniel E. Seavy, Diana L. Humple, Renée L. Cormier, Thomas Gardali

**Affiliations:** PRBO Conservation Science, Petaluma, California, United States of America; University of Plymouth, United Kingdom

## Abstract

The migratory biology and connectivity of passerines remains poorly known, even for those that move primarily within the temperate zone. We used light-level geolocators to describe the migratory geography of a North American temperate migrant passerine. From February to March of 2010, we attached geolocator tags to 33 Golden-crowned Sparrows (*Zonotrichia atricapilla*) wintering on the central coast of California, USA, and recovered four tags the following winter (October to December 2010). We used a Bayesian state-space model to estimate the most likely breeding locations. All four birds spent the breeding season on the coast of the Gulf of Alaska. These locations spanned approximately 1200 kilometers, and none of the individuals bred in the same location. Speed of migration was nearly twice as fast during spring than fall. The return rate of birds tagged the previous season (33%) was similar to that of control birds (39%), but comparing return rates was complicated because 7 of 11 returning birds had lost their tags. For birds that we recaptured before spring migration, we found no significant difference in mass change between tagged and control birds. Our results provide insight into the previously-unknown breeding provenance of a wintering population of Golden-crowned Sparrows and provide more evidence of the contributions that light-level geolocation can make to our understanding of the migratory geography of small passerines.

## Introduction

Bird migration presents a challenge for conservation and the study of avian ecology: no matter how well studied or protected a population may be during any one period in their annual cycle, most migratory birds disappear to unknown locations for the other periods of their lives. Not only does the reproductive success and/or survival during one period directly influence the number of birds in the next [1], [2], but conditions experienced by an individual in one season and location can influence their reproductive success or survival in the next (carry-over effects) [3], [4], [5], [6]. Population dynamics may be further complicated by spatial population structuring between breeding and non-breeding areas (migratory connectivity) [7], [8]. Finally, the conditions an individual encounters during the relatively brief period of actual migratory movement may be critically important [9].

Approximately half of the world's birds (5893 of 9930 species) are passerines and approximately 24% of these are migratory [10]. Unfortunately, the ability to study year-round life history of migratory passerines in their entire geographic context has been limited. Unlike with other groups of birds, such as waterfowl and shorebirds [7], [11], [12], [13], for passerines, the utility of banding recoveries to describe migratory connectivity has been limited because long-distance recaptures are rare [14]. Radio-tagging has been used to describe the migratory geography of shorebirds and waterfowl [15], [16], but this technique is less appropriate for passerines because they do not aggregate in large numbers where tagged individuals can be more easily be located, and because long-lasting batteries are too heavy to be carried by small birds . Satellite tags used for larger birds [17] are currently too heavy for passerines. Recent advances with stable isotopes and genetic fingerprinting have begun to provide more information about migratory connectivity, but at a resolution typically too coarse to adequately inform the fine spatial resolution desired of most ecological studies [18], [19], [20].

It is only with the recent development of small enough light-level-logging geolocator tags (hereafter, geolocators) that opportunities to study the complete migratory geography for passerines and other small birds have become available [14], [21], [22]. To date, the use of geolocators on passerines has been limited to tracking temperate-tropical migrants from their breeding grounds to their tropical wintering grounds and back [14], [21], [22], [23], [24]. Already, these efforts have provided novel information on the migratory geography of these species. In Wood Thrushes (*Hylocichla mustelina*), northward migrations to the breeding grounds were faster than southward migrations to non-breeding grounds, and these birds also demonstrated a high degree of migratory connectivity [21]. The same pattern of a faster spring migration than fall migration was also found with Veeries (*Catharus fuscescens*) and Red-backed Shrikes (*Lanius collurio*) [23], [24]. However, the degree to which these generalizations may apply to other passerine species, including those that winter at temperate latitudes and migrate to northern breeding grounds, remains untested.

We used geolocators to map the migratory geography of Golden-crowned Sparrows (*Zonotrichia atricapilla*) that winter in central coastal California, USA. Compared to temperate-tropical migrants, the migratory geography of passerines that migrate within the temperate latitudes has received much less attention. Indeed, even the basic breeding ecology of these species often remains poorly understood; the Golden-crowned Sparrow is virtually unstudied on its breeding grounds [25].

Our objectives were to (1) evaluate the utility of geolocators in providing information about the migratory geography of a population of Golden-crowned Sparrows that winters in central coastal California, (2) determine their provenance from across their breeding range in western Canada and Alaska [25], (3) determine if our wintering population consisted of birds from one or multiple breeding regions, and (4) evaluate the potential effects of the tags on these birds. With the recovery of four geolocator tags, we were able to demonstrate the provenance of these individuals and the utility of geolocators in providing information about the speed and timing of migration.

## Methods

### Banding returns

We assessed banding and encounter data to determine if any records connect breeding and wintering sites for (1) Golden-crowned Sparrows banded at the Palomarin Field Station, hereafter Palomarin, (1966–2010; latitude 37.93°, longitude −122.74°), and (2) all Golden-crowned Sparrows banded in North America (1922–2009; data provided by the U.S. Geological Survey Bird Banding Lab [USGS BBL]). We used date ranges of 1 June–10 August for breeding [25] and 15 November–1 April for wintering (based on Palomarin banding data) to ensure that banding and recovery locations would represent breeding and wintering areas, as opposed to migration.

### Study site and field methods

All birds were trapped in Bolinas, Marin County, California at study sites at or near Palomarin, a long-term study site in the Point Reyes National Seashore operated by PRBO Conservation Science. The vegetation at Palomarin is characterized by a mix of coastal scrub (dominated by coyote brush, *Baccharis pilularis*), Douglas-fir (*Pseudotsuga menziesii*), and mixed evergreen forest (dominated by Douglas-fir and coast live oak, *Quercus agrifolia* and California bay, *Umbellularia californica*). A second study site (4 km from Palomarin) on private land was dominated by a mix of native and non-native shrubs, especially Himalayan blackberry (*Rubus discolor*).

All birds were captured in baited Potter traps between 3 February and 2 March 2010. We attached geolocators to 33 Golden-crowned Sparrows. Geolocators record and store light-level data that can be downloaded when the bird is recaptured in order to interpolate latitude and longitude via sunrise and sunset timing [21]. We used Mk10S geolocators developed by British Antarctic Survey (BAS) with 15 mm long stalks positioned at a 30° angle. We attached these to birds with a leg-loop harness [26] of Kevlar 450 thread. The average weight of the harness and tag together was 1.1 g, 3–4% of the bird's weight. Each bird was banded with a federal aluminum band and a color band. We determined age of each bird, assessed the amount of subcutaneous fat, measured unflattened wing chord to the nearest mm, and weighed each bird to the nearest 0.1 g before and after the geolocator tag was attached. We were not able to sex the birds because Golden-crowned Sparrows are sexually monomorphic, and the results of genetic testing from pulled rectrices were inconclusive.

We captured an additional 28 control Golden-crowned Sparrows and collected all of the same data, fitting each individual with aluminum and color bands but no geolocator tag. When control and tagged birds were recaptured prior to spring migration, we reweighed each bird to evaluate short-term changes in body mass. Upon recapturing birds after they returned from their breeding grounds, we removed the geolocator tag if present and collected fat and mass data. We recaptured returning birds as soon as possible after first observing them.

### Ethics statement

Capture and handling followed strict bird safety protocols in accordance with the North American Banding Council [27]. All banding and tagging was approved by the USGS BBL (Permit Number: 09316).

### Statistical Analysis

To evaluate the short-term effect of tag attachment on Golden-crowned Sparrows, we used a repeated measures ANOVA to compare the average weight of tagged birds at their first capture (when geolocators were attached) to their weight at a subsequent capture (5–34 days after geolocators were attached) to the average weight of control birds at their first and subsequent (3–29 days later) capture. We used the significance of the interaction term, which describes a change in mass from the first to subsequent capture that was different between tagged and control birds, to test for an effect of tagging. After recovering the geolocators, we used the British Antarctic Survey (BAS) BasTrak software to decompress data from each geolocator into light files. Using this software, we corrected for clock drift that occurred during deployment. To convert the light files into locations, we did not use the BasTrak software. Instead, we analyzed the light data using the functions in the tripEstimation package [28], [29] in R (R version 2.10.1 [30]). Unlike the BasTrak software, which uses a threshold approach to estimate day length, the tripEstimation package uses the template fit method [31]. The template fit method has been demonstrated to have superior performance to the threshold method [31]. Furthermore, the tripEstimation package reduces the scatter in estimated locations by constraining the locations estimates with a land mask, the known locations of release and recovery, and the spatial boundaries beyond which locations are unrealistic; and then uses a state-space model (Kalman filter) to estimate the most likely positions for each twilight period [28], [29]. The advantages of this approach include reduced scatter in the location estimates and the ability to estimate locations during the equinoxes, (though with greater uncertainty than during the rest of the year) [28], [32].

For our analysis, we began by visually inspecting the daily light records and discarding all noisy twilight transitions. We used the first 20 transitions from each tag to generate a separate calibration for each tag. Because we used a post-deployment calibration, we assumed that tagged birds remained in the immediate vicinity of their release for the first two weeks after tagging. Observations and captures of tagged individuals in the immediate vicinity of their release supported this assumption.

We constrained possible locations with a land mask, the known locations of release and recovery, and the spatial boundaries beyond which we considered locations unrealistic (values outside of latitude 30 to 70 or longitude −170 to −100). This estimation requires light parameters and a movement model (for the state space model). For the light parameters, we used variance in light data = 7, variance in light attenuation = 10, an Ekstrom range of −10 to 8, and variance outside this range = 7. These values were similar to those used in work with BAS Mk9 geolocators [32]. For our movement model, we used a log normal distribution with a mean of 1 km/h and variance of 1. This movement model allows movements up to and beyond 250 km per 12 h day, which is consistent with previous estimates of migration speed using geolocators [21].

Using these parameters, for each tag we started by drawing 100,000 samples for burn-in and tuning of the proposal distribution. After this burn-in sample, we evaluated chain convergence by comparing the locations from the end of the burn-in sample to the locations after an additional run of 100,000 samples. Because there was almost no shift in the locations between these two samples, we assumed that the chains had converged. A final draw of 10,000 samples was then generated to describe the posterior distribution. We used the mean of the posterior sample as our estimate of the most likely location. We evaluated the timing of migration assuming that birds finished migration when latitude stabilized (using longitude to identify the end of migration yielded similar results). We plotted the locations between the end of spring migration and the beginning of fall migration and refer to these as the breeding season locations.

## Results

### Connectivity established from banding data

Of the 5251 Golden-crowned Sparrows banded at Palomarin between 1966 and 2010, only six were encountered away from their original banding site: none within the breeding range and only two outside of California (both during migration: one banded 5 November 1966 and recovered in coastal Washington in May 1967; and the second banded 18 October 1997 and recovered in northwestern Oregon on 20 April 1998). Of the 6452 Golden-crowned Sparrow band encounters reported for North America (USGS BBL, unpubl. data), only a single record definitively links breeding and wintering locations, a bird banded in central Alaska (latitude 63.58°, longitude −149.58°) by the Institute for Bird Populations on 25 June 1997 and recovered in inland southwestern Oregon (latitude 42.25°, longitude −123.25°) on 18 March 1999.

### Effects of geolocators on body mass and return rates

**Figure 1:**
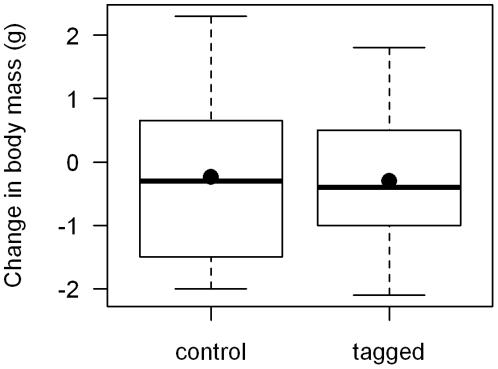
Short-term changes in body mass of Golden-crowned Sparrows with and without geolocator tags. The difference between initial capture and post-tagging (3–80 days) weights for Golden-crowned Sparrows with geolocator tags and metal and plastic leg bands (tagged; n = 10) and with metal and plastic leg bands only (control, n = 6). Box plots show the range (whiskers), interquatile range (box), median (horizontal line), and mean (dot) for each group. The average difference in pre- and post-tagging weights was not statistically significant (two-way repeated-measures ANOVA, interaction between tag attachment (yes/no) and capture time (before/after): F_1,15_ = 0.0068, p = 0.94; Fig. 1).

Prior to spring migration, we recaptured 10 of the tagged birds and 6 of the control birds from 3–34 days after they were originally tagged or (for control birds) banded and released without a geolocator. During this time, the mean change in weight of control birds was −0.24 g and the mean change in the weight of tagged birds was −0.30 g) were not statistically significant (two-way repeated-measures ANOVA, interaction between tag attachment (yes/no) and capture time (before/after): F_1,15_ = 0.0002, p = 0.94; Fig. 1).

The following year, we recaptured 11 of the previously tagged individuals (33% of 33) and 11 of the untagged control birds (39% of 28). Of the tagged group, 7 had lost their geolocators. The original weights of the 4 tagged sparrows that later returned with geolocators (mean = 33.3 g, SD = 0.6, n = 4) were similar to the original weights of the tagged individuals that did not return and those that returned but lost their tags (mean = 33.1 g, SD = 2.6, n = 29); and the birds returning with tags were neither the largest nor smallest individuals we tagged. All birds were tagged within a 28 day period (between 3 February and 2 March 2010); date of initial recapture during the return winter ranged from 5 October to 26 February for control birds, 9 October to 2 March for previously tagged birds who had lost their tags, and 19 October to 27 December for tagged birds.

### Migration timing and location of breeding grounds from recovered geolocators

**Figure 2:**
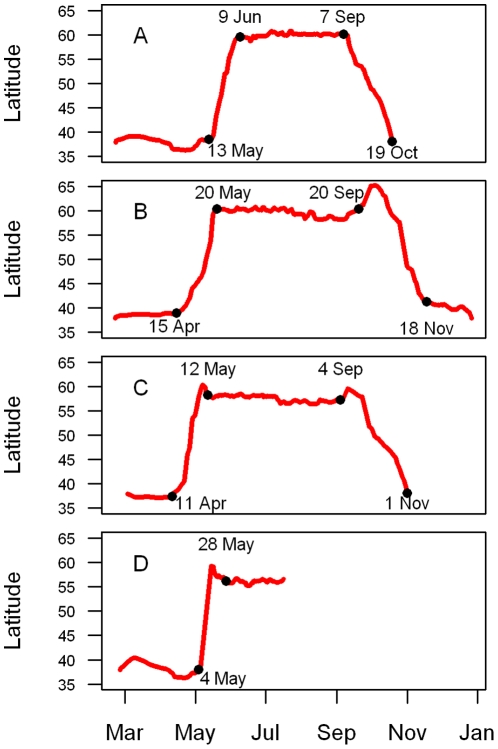
Migratory chronology of Golden-crowned Sparrow tagged in the winter/spring 2010 and recovered in fall/winter of 2010–11. The posterior mean estimate for the latitude of four Golden-Crowned Sparrows as a function of time. Dates indicate our estimates of the duration of spring and fall migration; the letters in each panel identify the four individual birds.

All four geolocators we recovered had successfully recorded light measurements, except data from one tag was unusable during fall migration, perhaps due to shading from feathers following molt. The four Golden-crowned Sparrows departed the wintering area between 11 April and 13 May and arrived on the breeding grounds between 12 May and 9 June (Fig. 2). The average length of the northward migration was 29 days (range 24–35 days, n = 4). In all cases, northward migration followed the Pacific coastline. The three individuals for which the tags collected data throughout the duration of their deployment initiated their southward migration between 4 and 20 September (Fig. 2), and arrived back at the wintering area between 19 October and 18 November (Fig. 2), such that the average length of the southward migration averaged 53 days (range 42–59 days, n = 3). Southward migration paths also appeared to follow the coast.

Despite our limited sample size, patterns in migration timing, distance, and duration were intriguing. Individuals that departed from the winter grounds later spent fewer days on northbound migration than individuals who left earlier. However, time spent migrating was not obviously related to migration distance or to time spent on breeding grounds. For the three birds whose tag data spanned the entire breeding season, the individual that spent the longest time on the breeding grounds (123 d) had the longest spring and fall migrations, and the individual that spend the shortest time on the breeding grounds (90 d) had the shortest migrations.

**Figure 3:**
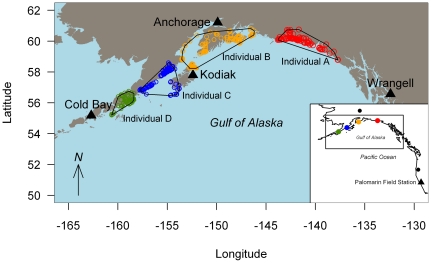
Breeding locations of Golden-crowned Sparrows that wintered near the Palomarin Field Station, Marin County, California. Inset: Location of Palomarin Field Station where Golden-crowned Sparrows were tagged during the winter and the approximate breeding locations from the four geolocators that were recovered. The black circle marks the breeding and wintering location of the one band recovery for the wintering and breeding periods. Main map: Circles mark the posterior mean estimate of locations (1 per day) during the breeding season for each of the four individuals. The polygons are minimum convex hulls around the points for a single individual.

All four Golden-crowned Sparrows spent the breeding season along the coast of the Gulf of Alaska (Fig. 3). The locations ranged (east to west) from (1) the Alaskan Panhandle just north of Wrangell, (2) the Anchorage area, (3) Kodiak Island, and (4) the Alaskan Peninsula (Fig. 3). The distance along the coast between the eastern and westernmost breeding locations was approximately 1200 km; and none of the polygons encompassing all locations during the breeding season for each of the four individuals overlapped (Fig. 3).

## Discussion

All four Golden-crowned Sparrows whose geolocator tags were recovered following their round-trip migration to their breeding grounds went to the same general region: the Gulf of Alaska coastline. Despite the small sample size, this suggests the Gulf of Alaska to be a key breeding area for sparrows wintering in central coastal California. The results however did not indicate very strong migratory connectivity in that, despite sharing the same wintering grounds, no two birds bred in the same area within the Gulf of Alaska. Moreover, the distance between the eastern- and western-most individuals was approximately 1200 km (equivalent to the length of the entire California coastline). Other geolocator studies of small landbirds have revealed patterns of both strong (three passerines [14], [21]) and weak (one near-passerine, the Hoopoe [*Upupa epops*] [22]) connectivity. Perhaps the most similar pattern of connectivity to Golden-crowned Sparrows has been the case of the Veery (*Catharus fuscescens*), in which five individuals tagged in Delaware, USA, all initially settled in Brazil, south of the Amazon River, but in geographically separate areas [23]. As more geolocator data are generated, we propose that there will be a need to develop quantitative measures of migratory connectivity.

Further study is needed to determine if, conversely, most birds breeding in the Gulf of Alaska winter in central coastal California; to enhance our knowledge of breeding provenance from this wintering region; and to determine the breeding provenance from other regions. Interestingly, the only definitive band recovery to connect a breeding Golden-crowned Sparrow to its wintering grounds revealed an inland-breeding bird (central Alaska) wintering also at an inland locale (southwestern Oregon). Our data also revealed a predominantly coastal migration route for this Golden-crowned Sparrow population, and indicate that spring migration to the breeding grounds takes nearly half the time as fall migration. Spring migration in Wood Thrushes, Veeries and Red-backed Shrikes was also faster than fall migration [21], [23], [24].

Several limitations should be recognized in the interpretation of our results. First, we relied on a post-deployment calibration from a short period on the wintering grounds. If the vegetation structure or topography at the breeding grounds was dramatically different, this may have affected our estimate of the breeding locations. This limitation is true for most geolocator studies [33]. Additionally, we did not conduct a post-deployment calibration. If there was a significant shift in the sensitivity of the light level logger, this could also influence our location estimates. However, because we did not see a pronounced drift in the latitude estimates during the course of the breeding season (Fig. 2), this seems unlikely. Finally, the state-space method we used to filter the location estimates requires specifying a movement model to describe the likely distances that an individual would travel in a day [28]. Currently, the method can only accommodate a single movement model, but a model in which daily movement rates are lower during the breeding and wintering season and greater during migration would certainly be more realistic. While the consequences of assuming a single movement model are not currently understood [28], we expect that this assumption would have a greater impact on the estimate of the migration period locations than on the breeding period locations.

We investigated the effects of tags at two temporal scales. First, we examined changes in body mass over the first few months after trapping (before birds migrated north). During this period we found little evidence that the mass of tagged birds declined more than control birds. Second, we compared the return rate of tagged birds (33%) to control birds (39%) the following fall, although the return rate includes individuals who lost their tags and timing of tag loss is unknown. Again, the similarity in these rates provides little evidence of a large tag effect, and a similar return rate result was also found in Gray Catbirds [14]. Furthermore, there was no evidence that the birds that returned were simply the largest of the birds that we tagged and thus better able to handle the tag. However, given that our sample sizes were small, and the demonstrated effects that these tags have on aerodynamics [34], our results should be interpreted cautiously and we encourage researchers to continue to evaluate the effects that tags may have on body condition, survival, and behavior.

Seven Golden-crowned Sparrows returned to their wintering grounds with tags and harness missing. We strongly suspect that these individuals were successful at removing their tags simply by picking at the fibrous Kevlar thread harness, as indicated by the condition of harnesses on recaptured tagged birds during both seasons. Hence, we suggest that alternate harness materials be used for this (and likely other) thick-billed species.

Knowledge of breeding origin may shed light on observed demographic patterns of wintering Golden-crowned Sparrows and other species, and direct further research questions. This includes examining impacts of weather patterns and global climate change on both wintering and breeding populations. With the advent of small geolocator tags, opportunities now exist to examine migratory geography in other temperate-wintering migratory passerines, as well as in other small birds, either in the absence of band returns [22] or by combining band returns with geolocator information [14]. Knowledge of migratory geography in passerines and other small species is likely to expand exponentially in the coming years and direct conservation and research activities.
